# Fermented Oyster Extract Attenuated Dexamethasone-Induced Muscle Atrophy by Decreasing Oxidative Stress

**DOI:** 10.3390/molecules26237128

**Published:** 2021-11-25

**Authors:** Seyeon Oh, Chang Hu Choi, Bae-Jin Lee, Joung-Hyun Park, Kuk-Hui Son, Kyunghee Byun

**Affiliations:** 1 Functional Cellular Networks Laboratory, Department of Medicine, Graduate School and Lee Gil Ya Cancer and Diabetes Institute, Gachon University College of Medicine, Incheon 21999, Korea; seyeon8965@gmail.com; 2 Department of Thoracic and Cardiovascular Surgery, Gachon University Gil Medical Center, Gachon University, Incheon 21565, Korea; cch624@gilhospital.com; 3 Marine Bioprocess Co., Ltd., Smart Marine BioCenter, Busan 46048, Korea; hansola82@hanmail.net (B.-J.L.); pdc327@hanmail.net (J.-H.P.); 4 Department of Anatomy and Cell Biology, Gachon University College of Medicine, Incheon 21936, Korea

**Keywords:** muscle atrophy, GABA, lactate, oxidative stress, Nrf2

## Abstract

It is well known that oxidative stress induces muscle atrophy, which decreases with the activation of Nrf2/HO-1. Fermented oyster extracts (FO), rich in γ-aminobutyric acid (GABA) and lactate, have shown antioxidative effects. We evaluated whether FO decreased oxidative stress by upregulating Nrf2/HO-1 and whether it decreased NF-κB, leading to decreased IL-6 and TNF-α. Decreased oxidative stress led to the downregulation of Cbl-b ubiquitin ligase, which increased IGF-1 and decreased FoxO3, atrogin1, and Murf1, and eventually decreased muscle atrophy in dexamethasone (Dexa)-induced muscle atrophy animal model. For four weeks, mice were orally administered with FO, GABA, lactate, or GABA+Lactate, and then Dexa was subcutaneously injected for ten days. During Dexa injection period, FO, GABA, lactate, or GABA+Lactate were also administered, and grip strength test and muscle harvesting were performed on the day of the last Dexa injection. We compared the attenuation effect of FO with GABA, lactate, and GABA+lactate treatment. Nrf2 and HO-1 expressions were increased by Dexa but decreased by FO; SOD activity and glutathione levels were decreased by Dexa but increased by FO; NADPH oxidase activity was increased by Dexa but decreased by FO; NF-κB, IL-6, and TNF-α activities were increased by Dexa were decreased by FO; Cbl-b expression was increased by Dexa but restored by FO; IGF-1 expression was decreased by Dexa but increased by FO; FoxO3, Atrogin-1, and MuRF1 expressions were increased by Dexa but decreased by FO. The gastrocnemius thickness and weight were decreased by Dexa but increased by FO. The cross-sectional area of muscle fiber and grip strength were decreased by Dexa but increased by FO. In conclusion, FO decreased Dexa-induced oxidative stress through the upregulation of Nrf2/HO-1. Decreased oxidative stress led to decreased Cbl-b, FoxO3, atrogin1, and MuRF1, which attenuated muscle atrophy.

## 1. Introduction

It is known that excessive oxidative stress caused by an imbalance between the generation and removal of intracellular reactive oxygen species (ROS) can decrease protein synthesis and enhance protein degradation, eventually leading to muscle atrophy [1,2,3].

Dexamethasone (Dexa) can cause oxidative stress in various cells such as skeletal muscle cells, adipocytes, pancreatic cells, and osteoblastic cells [4,5,6,7,8]. Excessive ROS in rodent myotubes has reportedly enhanced expression of the ubiquitin ligase casitas B-lineage lymphoma proto-oncogene-b (Cbl-b), eventually leading to the degradation of insulin receptor substate-1 (IRS-1) which causes muscle atrophy [9].

In addition, the upregulation of Cbl-b by ROS leads to decreased insulin-like growth factor-1 (IGF-1) signaling [9,10], which leads to increased dephosphorylation of Forkhead box O (FoxO) 3a and consequently induces upregulation of muscle atrophy-associated ubiquitin ligases, such as muscle atrophy F-box (MAFbx)/atrogin-1 and muscle RING finger 1 (MuRF1) [11].

Nuclear factor erythroid 2-related factor 2 (Nrf2) has essential protective roles against oxidative stress-induced muscle damage [12] and atrophy [13].

In the homeostatic state without oxidative stress, inactivated Nrf2 exists in the cytoplasm by binding to Kelch-like ECH-associated protein 1 (Keap1), which prohibits the nuclear translocation of Nrf2 [12,14,15].

Under oxidative stress, the binding of Nrf2 to Keap1 is broken, leading to the translocation of Nrf2 into the nucleus, consequently stimulating the transcriptional activity of antioxidant response element (ARE) response genes. ARE response genes induce the upregulation of phase II antioxidant enzymes such as heme oxygenase-1 (HO-1), glutathione peroxidase-1 (GPx1), NAD(P)H quinone dehydrogenase-1 (NQO-1), and thioredoxin-1 (Trx1) [13,14,15]. Glutathione (GSH) is a nonenzymatical antioxidant that reacts with superoxide, nitric oxide, hydroxyl radical, and peroxynitrite [16]. GSH is also increased by upregulation of Nrf2 and HO-1 [17]. Expression of superoxide dismutase (SOD), which is also a ROS scavenger, is also increased by HO-1 [18].

HO-1 has an antioxidant defense mechanism that works by breaking down heme into carbon monoxide, free iron, and biliverdin. Biliverdin is further broken down into bilirubin, which has the antioxidant potential [19,20]. In addition, bilirubin is known to inhibit the activity of NADPH oxidase, which generates ROS [21].

ROS are generated by mitochondria, NADPH oxidase, and the endoplasmic reticulum, leading to the activation and translocation of NF-κB. When translocated to the nucleus, NF-κB enhances various target gene transcription, such as tumor necrosis factor-alpha (TNF-α), interleukin (IL)-1β, and IL-6 [22]. As inflammatory cytokines, TNF-α, IL-1β, and IL-6 all have the central role in mediating muscle wasting and atrophy [23].

Several oyster extracts such as hydrolysates of oyster (*Saccostrea cucullata*) protein have shown antioxidant effects or free radical scavenging ability [24]. The phenolic compounds from the Pacific oyster (*Crassostrea gigas*) have even attenuated the apoptosis of hepatocytes by oxidative stress [25,26,27].

By fermentation with *Lactobacillus brevis* BJ20, fermented oyster extracts (FO) from *Crassostrea gigas* have an increased concentration of γ-aminobutyric acid (GABA), which is bio-converted from glutamic acid within the oyster and creates high amounts of lactate [28,29].

FO showed antioxidant activities and decreased oxidative cell injuries in oblasts by increasing the activation of Nrf2/HO-1 signaling [30]. Dexa is frequently used for treating respiratory diseases such as chronic obstructive pulmonary disease or asthma [31,32]. It is also used for various inflammatory diseases, autoimmune diseases, and even cancer [31,32]. It is also known that muscle atrophy induced by Dexa is associated with increased morbidity and mortality after Dexa therapy. Thus many studies have been performed to find new targets for inhibiting Dexa-induced muscle atrophy [33,34,35].

Even though muscle atrophy is closely related to oxidative stress, and FO is known to have antioxidative effects by activating the Nrf2/HO-1 signal, it is still unknown whether FO could attenuate muscle atrophy by modulating the Nrf2/HO-1 signal. We hypothesized that FO would (1) decrease oxidative stress by upregulating Nrf2/HO-1 and (2) decrease NF-κBwhich, which would, in turn, decrease IL-6 and TNF-α. We also hypothesized that decreased oxidative stress would lead to the downregulation of Cbl-b, which would increase IGF-1 and decrease FoxO3, atrogin1, and Murf1, eventually decreasing muscle atrophy. In our study, we evaluated the attenuation effect of FO on muscle atrophy in the Dexa-induced muscle atrophy animal model and compared the attenuation effect of FO with GABA and lactate.

## 2. Results

### 2.1. FO Led to Increased Nrf2/HO-1, Decreased NADPH Activity, and Decreased OXIDATIVE Stress in Muscle of Dexa-Treated Animals

**Figure 1:**
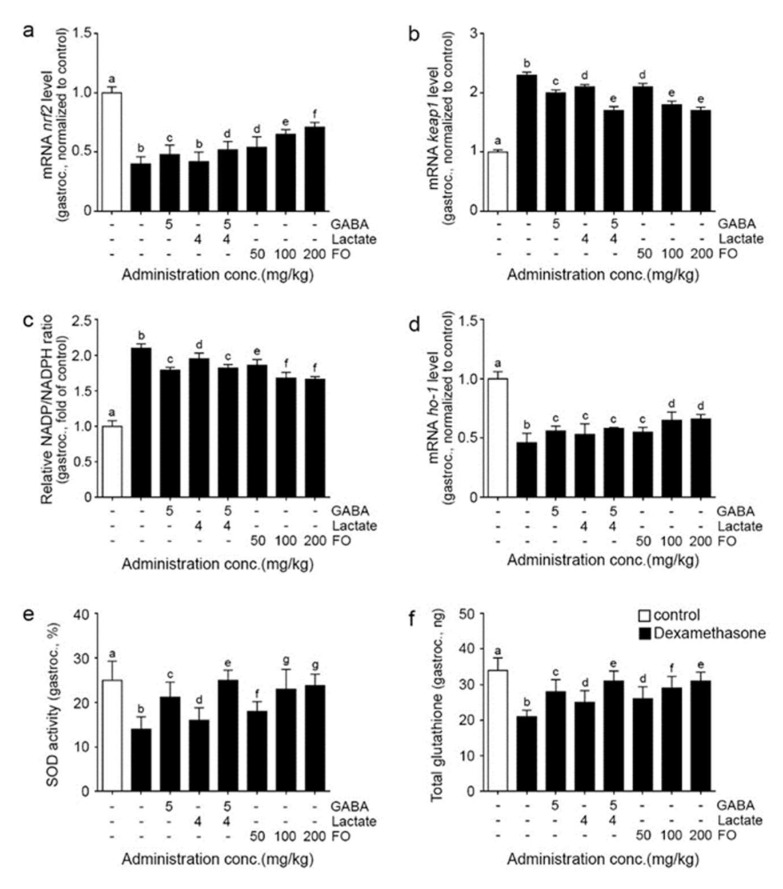
FO reduced ROS through Nrf2/Ho-1 in the gastrocnemius muscle atrophy induced mouse model; (**a**) The mRNA levels of nrf2 were decreased by Dexa/Saline but increased by GABA, lactate, GABA+lactate, and FO treatment; (**b**) The mRNA levels of keap1 were increased by Dexa/Saline but decreased by GABA, lactate, GABA+lactate, and FO treatment; (**c**) Relative NADP/NDPH ratios were increased by Dexa/Saline but decreased by GABA, lactate, GABA+lactate, and FO treatment; (**d**) The mRNA levels of ho-1 were decreased by Dexa/Saline but increased by GABA, lactate, GABA+lactate, and FO treatment; (**e**,**f**) SOD activity (**e**) and total glutathione (**f**) were decreased by Dexa/Saline but increased by GABA, lactate, GABA+lactate, and FO treatment. Different letters, a–g, indicate significant differences among the group as determined by multiple comparisons (Mann-Whitney U test); *p* < 0.05; fermented oyster extracts (FO); γ-aminobutyric acid (GABA); heme oxygenase (ho-1); gastrocnemius (gastroc.); kelch-like ECH-associated protein 1 (keap1); nicotinamide adenine dinucleotide phosphate (NADP); nicotinamide adenine dinucleotide phosphate hydrate (NADPH); nuclear factor erythroid-2-related factor 2 (nrf); superoxide dismutase (SOD).

Nrf2 mRNA expression was significantly decreased in the gastrocnemius muscle after Dexa treatment and significantly increased after the administration of GABA, GABA+lactate, and FO. The most notable increase was seen with 200 mg/kg of FO (Figure 1a).

Keap1 mRNA expression was significantly increased in the gastrocnemius muscle after Dexa treatment and significantly decreased by administering GABA, lactate, GABA+lactate, and FO. The most prominent decrease was seen with GABA+lactate and 100 and 200 mg/kg of FO (Figure 1b).

NADP/NADPH ratio was significantly increased in the gastrocnemius muscle after Dexa treatment and significantly decreased after using GABA, lactate, GABA+lactate, and FO. The most prominent decrease was seen with 100 and 200 mg/kg of FO (Figure 1c).

HO-1 mRNA expression was significantly decreased in the gastrocnemius muscle after Dexa treatment and significantly increased after the administration of GABA, lactate, GABA+lactate, and FO. The most notable increase was seen with 100 and 200 mg/kg of FO (Figure 1d).

SOD activity was significantly decreased in the gastrocnemius muscle after Dexa treatment and significantly increased after administering GABA, lactate, GABA+lactate, and FO. The most notable increase was seen with GABA+lactate (Figure 1e).

The amount of total glutathione was significantly decreased in the gastrocnemius muscle after Dexa treatment and significantly increased after the administration of GABA, lactate, GABA+lactate, and FO. The most significant increase was seen with GABA+lactate and 200 mg/kg of FO (Figure 1f).

### 2.2. FO Decreased Expression of NF-κB/IL-6/TNF-α

**Figure 2:**
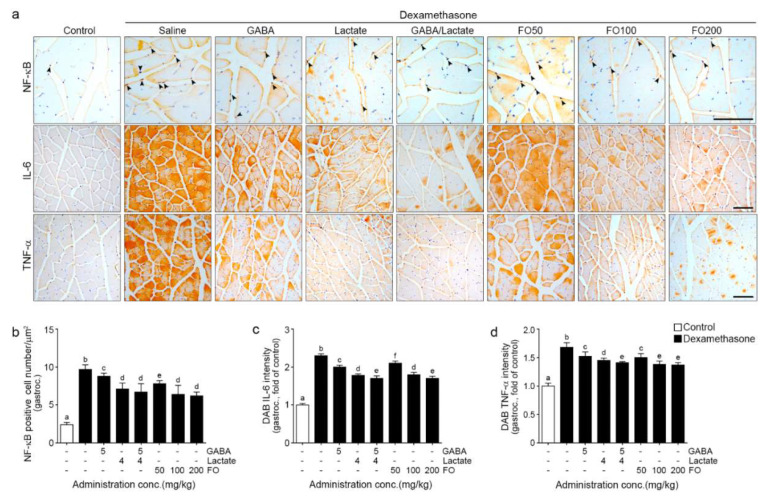
FO reduced intramuscular inflammation in a muscle atrophy-induced mouse model; ((**a**) first line and (**b**)) The number of NF-κB translocated into the nucleus was increased Dexa/Saline but decreased by GABA, lactate, GABA+lactate, and FO treatment; ((**a**), first and second line, (**c**,**d**)) The intensity of IL-6 and TNF-α were increased Dexa/Saline but decreased by GABA, lactate, GABA+lactate, and FO treatment. Scale bar = 100 μm. Different letters a–f indicate significant differences among groups as determined by multiple comparisons (Mann-Whitney U test); *p* < 0.05., 3,3′-Diaminobenzidine tetrahydrochloride (DAB); fermented oyster extracts (FO); γ-aminobutyric acid (GABA); gastrocnemius (gastroc.); interleukin-6 (IL-6); nuclear factor kappa-light-chain-enhancer of activated B cells (NF-κB); tumor necrosis factor-alpha (TNF-α).

NF-κBexpression in the nuclei was significantly increased by Dexa and significantly decreased by GABA, lactate, GABA+lactate, and FO. The most prominent decrease was seen with lactate, GABA+lactate, and 100 and 200 mg/kg of FO (first line in Figure 2a,b).

IL-6 expression was significantly increased by Dexa and significantly decreased by GABA, lactate, GABA+lactate, and FO. The most prominent decrease was seen with GABA+lactate and 200 mg/kg of FO (second line in Figure 2a,c).

TNF-α expression was significantly increased by Dexa and significantly decreased by GABA, lactate, GABA+lactate, and FO. The most prominent decrease was seen with GABA+lactate and 100 and 200 mg/kg of FO (third line in Figure 2a,d).

### 2.3. FO Leads to Decreased Expression of Cbl-b, Increased IGF-1, and Decreased FoxO3/Atrogin-1/Murf-1 in the Muscle of Dexa-Treated Animals

**Figure 3:**
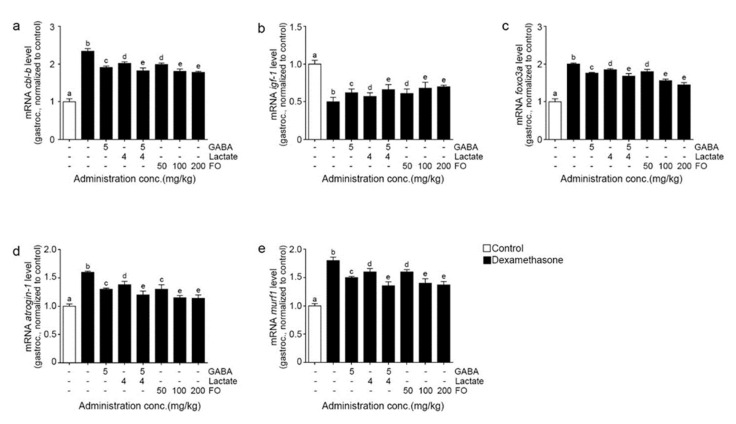
FO reduced muscle proteolysis through IGF-1 in a muscle atrophy-induced mouse model. (**a**) The mRNA levels of cbl-b were increased by Dexa/Saline but decreased by GABA, lactate, GABA+lactate, and FO treatment; (**b**) The mRNA levels of igf-1 were decreased by Dexa/Saline but increased by GABA, lactate, GABA+lactate, and FO treatment; (**c**–**e**) The mRNA levels foxo3a (**c**), atrogin-1 (**d**), and murf1 (**e**), were increased by Dexa/Saline but decreased by GABA, lactate, GABA+lactate, and FO treatment. a–e Different letters indicate significant differences among groups as determined by multiple comparisons (Mann-Whitney U test); *p* < 0.05. a muscle-specific F-box protein (atrogin-1); Cbl Proto-Oncogene B (cbl-b); fermented oyster extracts (FO); γ-aminobutyric acid (GABA); gastrocnemius (gastroc); forkhead box O3 (foxO3a); insulin-like growth factor 1 (igf-1); Muscle RING-finger protein-1 (murf1).

The mRNA levels of Cbl-b expression were significantly increased by Dexa and significantly decreased by GABA, lactate, GABA+lactate, and FO. The most prominent decrease was seen with GABA+lactate and 100 and 200 mg/kg of FO (Figure 3a).

The mRNA levels of IGF-1 expression were significantly decreased by Dexa and significantly increased by GABA, lactate, GABA+lactate, and FO. The most notable increase was seen with GABA+lactate and 100 and 200 mg/kg of FO (Figure 3b).

The mRNA levels of FoxO3a, atrogin-1, and murf1 expression were significantly increased by Dexa and significantly decreased by GABA, lactate, GABA+lactate, and FO. The most prominent decrease was seen with GABA+lactate and 100 and 200 mg/kg of FO (Figure 3c–e).

### 2.4. FO Attenuated Muscle Atrophy and Improved Grip Strength

**Figure 4:**
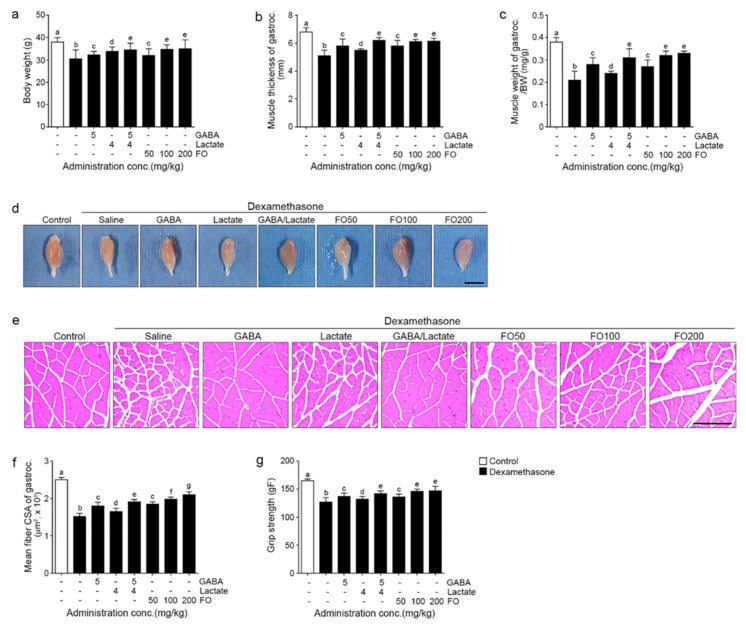
FO improved body weight, muscle thickness, weight, and fiber size of gastrocnemius and strength in a muscle atrophy induced mouse model. Bodyweight was decreased by Dexa/Saline but increased by GABA, lactate, GABA+lactate, and FO treatment (**a**). The muscle thickness of gastrocnemius (**b**–**d**) and muscle weight of gastrocnemius (**b**,**d**) were decreased by Dexa/Saline but increased by GABA, lactate, GABA+lactate, and FO treatment (**c**,**d**). The mean CSA of gastrocnemius muscle fiber was decreased Dexa/Saline but increased by GABA, lactate, GABA+lactate, and FO treatment. Scale bar = 1 cm (**e**,**f**). The grip strength was decreased Dexa/Saline but increased by GABA, lactate, GABA+lactate, and FO treatment. Scale bar = 100 μm (**g**). Different letters a–g indicate significant differences among the group as determined by multiple comparisons (Mann-Whitney U test); *p* < 0.05. BW, body weight; cross-sectional area (CSA); fermented oyster extracts (FO); γ-aminobutyric acid (GABA); gastrocnemius (gastroc.).

The bodyweight of the Dexa group was 0.80 times lower than that of the control group. It was significantly increased by GABA, lactate, GABA+lactate, 50, 100, and 200 mg/kg FO (1.1 times than Dexa group) (Figure 4a).

The thickness of the gastrocnemius of the Dexa treated group was 0.8 times lower than that of the control group. It was significantly increased by GABA (1.1 times), lactate (1.1 times), GABA+lactate (1.2 times), 50 mg/kg FO (1.1 times), 100 mg/kg FO (1.2 times), and 200 mg/kg FO (1.2 times). The most notable increase was seen with GABA+lactate and 100 and 200 mg/kg of FO (Figure 4b,d).

The weight (normalized by body weight) of the gastrocnemius of the Dexa treated group was 0.6 times lower than that of the control group. It was significantly increased by GABA (1.3 times), lactate (1.1 times), GABA+lactate (1.5 times), 50 mg/kg FO (1.3 times), 100 mg/kg FO (1.5 times), and 200 mg/kg FO (1.6 times). The most prominent increase was seen with GABA+lactate and 100 and 200 mg/kg of FO (Figure 4c).

The mean CSA of the muscle fibers of the Dexa group was 0.60 times lower than that of the control group. It was significantly increased by GABA (1.2 times than Dexa group), lactate (1.1 times), GABA+lactate (1.3 times), 50 mg/kg FO (1.2 times), 100 mg/kg FO (1.3 times), and 200 mg/kg FO (1.4 times). The most prominent increase was shown at 200 mg/kg FO (Figure 4e,f).

The grip strength of the Dexa group was 0.8 times lower than that of the control group. It was significantly increased by GABA (1.1 times), lactate (1.0 times), GABA+lactate (1.1 times), 50 mg/kg FO (1.1 times), 100 mg/kg FO (1.1 times), and 200 mg/kg FO (1.2 times). The most prominent decrease was seen with GABA+lactate and 100 and 200 mg/kg of FO (Figure 4g).

## 3. Discussion

Oxidative stress is one of the main pathophysiological mechanisms of muscle atrophy and is induced by various conditions such as malnutrition, disuse, cancer, diabetes, denervation, and aging [2,36,37].

Excessive intracellular ROS leads to massive protein oxidation, which enhances protein degradation by various ubiquitin-proteasome systems and downregulation of the phosphoinositide 3-kinase (PI3K)/Akt pathway, which is essential for protein synthesis [37].

ROS accumulation is known to be increased in aged satellite cells or proliferating aged myoblasts. Excessive ROS change mitochondrial function, which is the main mechanism in the development of primary sarcopenia induced by aging [38]. Moreover, Dexa -induced muscle atrophy is also related to increased ROS accumulation [39]. Glucocorticoid treatment increases the expression of NADPH oxidase, which generates ROS [40,41].

Disturbance of Nrf2-Keap1 signaling is accompanied by human aging and is associated with the development of sarcopenia. In addition, inhibition of Nrf2 by caveolin-1 enhances premature senescence [42]. It is also known that Nrf2 has protective roles regarding oxidative stress-induced muscle damage [12]. Sulforaphane (SFN), an inducer of Nrf2 activation, decreased muscle atrophy induced by oxidative stress [13].

On the other hand, GABA intake decreased oxidized proteins, which is increased in the skeletal muscle by high fat and increased expression of SOD, catalase, and glutathione peroxidase. Thus, GABA showed an antioxidant effect in the skeletal muscle [43].

Lactate is known to have antioxidant effects by modulating Nfr2 and reduced cell death which is induced by ROS [44]. Through the specialized fermentation process, the oyster extract has enriched GABA and lactate, increasing the oyster’s bioactive properties [45].

Here, we evaluated the attenuation effect of FO on Dexa-induced muscle atrophy by increasing the activity of Nrf2. Dexa treatment decreased Nrf2 expression, but this was restored by the administration of FO (Figure 1a). On the other hand, Keap1 expression was increased by Dexa, and it was decreased by FO (Figure 1b).

The NADP+/NADPH is frequently used for evaluating NADPH oxidase activity [46]. NADPH oxidases are known as the primary source of ROS in the skeletal muscle cells [47].

SOD transforms superoxide into H_2_O_2_ and O_2_ [48]. Deletion of SOD was reported to decrease muscle mass since SOD is essential for maintaining muscle fibers [49].

GSH is the most abundant nonprotein thiol in cells and acts as a nonenzymatic antioxidant that detoxifies hydroperoxides [50,51]. It is reported that a cystine-based GSH precursor attenuated decreasing muscle mass by reducing the production of ROS [52].

NADPH oxidase activity was increased by Dexa and decreased by FO most prominently at 100 and 200 mg/kg of FO (Figure 1c). HO-1 expression was significantly decreased by Dexa and significantly increased by FO. This increase was more evident in 100 and 200 mg/kg of FO than individual administration of GABA, lactate, or GABA+lactate (Figure 1d). The activity of SOD was significantly decreased by Dexa and significantly increased by FO. This increase was more evident in 100 and 200 mg/kg of FO (Figure 1e). The amount of glutathione was significantly decreased by Dexa and significantly increased by FO. This increase was more evident in GABA+lactate and 200 mg/kg of FO (Figure 1f). It seemed that FO increased Nrf2 and HO-1, which led to decreased oxidative stress by decreasing NAPDH oxidase activity, increasing SOD, and increasing GSH in the muscle.

The increased activation of NF-κB led to skeletal muscle atrophy through various mechanisms. NF-κB upregulates the expression of components of the ubiquitin-proteasome system, which causes degradation of specific muscle proteins [53,54]. NF-κB increased the expression of proinflammatory cytokines, such as IL-1β or IL-6, which directly or indirectly enhance muscle wasting [55]. Furthermore, NF-κB inhibits muscle differentiation-related genes, such as myoblast determination protein 1 and myogenin, which lead to the regeneration of atrophied skeletal muscle [56,57].

TNF-α is also related to muscle atrophy. C2C12 myotubes treated with TNF-α showed an increased Atrogin-1 and Murf-1 (part of the ubiquitin-proteasome system) and decreased muscle differentiation-related genes such as myoblast determination protein 1 and myogenin [58].

Our results showed that the activity of NF-κB and expression of IL-6 and TNF-α were increased by Dexa but significantly decreased by FO. The decreasing effect of 100,200 mg/kg of FO was similar to GABA+lactate (Figure 2).

IGF-1 is involved in various pathways related to muscle growth, differentiation, and regeneration [59]. It was reported that FO enhanced the release of IGF-1 and upregulated the down signal pathway of the IGF-1 receptor (involving GSK-3β at Ser9), leading to bone formation [60]. IGF-1 signaling is disturbed by ROS-induced upregulation of Cbl-b ubiquitin ligase [9,10]. Decreased IGF-1 signaling led to increased dephosphorylation of Foxo3a, which induces the expression of MAFbx1/atrogin-1 and MuRF-1 [11]. Dexa treatment reduced the diameter of C2C12 myotubes and increased Cbl-b ubiquitin ligase, atrogin-1, and Murf-1. Moreover, Dexa also decreased phosphorylated Foxo3a and increased total Foxo3a expression in C2C12 myotubes [39].

Herein, we evaluated whether the decreased ROS caused by Nrf2/HO-1 upregulation affects the expression of Cbl-b ubiquitin ligase and IGF-1. The expression of *cbl-b* ubiquitin ligase was increased by Dexa and decreased by FO, with the most decrease seen with GABA+lactate and 100 and 200 mg/kg of FO in gastrocnemius and soles Figure 3a and Figure S1a). The expression of IGF-1 in gastrocnemius and soles was decreased by Dexa and restored by FO. The increasing effects of GABA+lactate and 100 and 200 mg/kg of FO were not different (Figure 3b and Figure S1b). The expressions of foxO3a, atrogin-1, and murf1 in gastrocnemius and soles increased significantly by Dexa but significantly decreased by FO. The decreasing effects of GABA+lactate and 100 and 200 mg/kg of FO were not different (Figure 3c–e and Figure S1c–e).

The bodyweight, thickness, weight of the gastrocnemius and soleus, and grip strength were decreased by Dexa but restored by FO. The restoring effects of GABA+lactate and 100 and 200 mg/kg of FO were not different (Figure 4a–d,g, and Figure S2a–c). Moreover, the CSA of muscle fibers of gastrocnemius and soleus were increased by FO, with 200 mg/kg of FO being the most effective (Figure 4e,f and Figure S2d,e).

Recently, much evidence suggested that extreme loss of muscle mass and muscle function increase morbidity and mortality as well as disability in physical function [61].

Thus, interest in finding proper therapeutics for muscle atrophy related to various diseases has rapidly increased. In conclusion, we showed that FO decreased oxidative stress by upregulation of Nrf2/HO-1 and increased defense mechanisms such as GSH and activity of SOD in the Dexa treated muscle. Thus, the decreased oxidative stress induced to decreased activity of NF-κB, which plays a role in upregulating proinflammatory cytokines. In addition, the expression of Cbl-b was reduced by decreased oxidative stress and led to a restored expression of IGF-1, which consequently led to decreased FoxO3, atrogin1, and murf1. Thus, decreased inflammatory cytokines and muscle atrophy genes lead to attenuate muscle atrophy and decreased muscle function.

## 4. Materials and Methods

### 4.1. Preparation of FO, GABA, and Lactate

FO extract was obtained from Marine Bioprocess Co., Ltd. (Gijang, Busan, Korea). For extraction, FO was prepared by fermentation with *Lactobacillus brevis* BJ20 [29,45,62,63,64], and glutamic acid and dextrin were used on behalf of monosodium glutamate and an excipient, respectively. Glutamate was used as a precursor to producing GABA through a decarboxylation reaction for fermentation with *Lactobacillus brevis* BJ20 [45,62,63,64]. In this study, used FO was the same batch as used that in a previous report [45,62,63,64], which was composed of 46 g/100 g carbohydrate, 36 g/100 g crude protein, 6.3 g/100 g sugars, 114 mg/g GABA, and 40.3 mg/g lactic acid [45,64]. FO was diluted with saline to adjust the final treatment concentration before use.

### 4.2. Dexa Incued Muscle Atrophy Mice Model

Male ICR mice (aged nine weeks) were obtained from Orient Bio (Seongnam, Gyeonggi-do, Korea) and cared at the same condition (temperature of −23 °C, relative humidity of 50%, and a dark/light cycle of 12/12 h) with free access to rodent chow and water. After one week of the acclimation period, mice were then randomly categorized into eight groups like below:

(i) Control group: mice were orally administered with saline.
(ii) Dexa/Saline group: mice were orally administered with saline, and muscle atrophy was induced with dexamethasone.
(iii) Dexa/GABA group: mice were orally administered with 5 mg/kg of GABA, and muscle atrophy was induced with dexamethasone.
(iv) Dexa/Lactate group: mice were orally administered with 4 mg/kg of GABA, and muscle atrophy was induced with dexamethasone.
(v) Dexa/GABA+Lactate group: mice were orally administered with 5 mg/kg of GABA + 4 mg/kg of GABA, and muscle atrophy was induced with dexamethasone.
(vi) Dexa/FO50: mice were orally administered with 50 mg/kg of FO, and muscle atrophy was induced with dexamethasone.
(vii) Dexa/FO100: mice were orally administered 100 mg/kg of FO, and muscle atrophy was induced with dexamethasone.
(viii) exa/FO200: mice were orally administered 200 mg/kg of FO, and muscle atrophy was induced with dexamethasone.

FO, GABA, or Lactate was orally administrated once a day for four weeks. After four weeks of oral administration, 1 mg/kg of Dexa was subcutaneously injected once a day for ten days with oral administration [65,66,67]. After the last Dexa injection, the grip strength test was performed. All animal experiments were performed with approval according to the ethical principles of the Institutional Animal Care and Use Committee of Gachon University (approval no. LCDI-2020-0030).

### 4.3. RNA Extraction and Complementary DNA (cDNA) Synthesis and Quantitative Real-Time Polymerase Chain Reaction (qRT-PCR)

The mice gastrocnemius and soleus muscles were homogenized in ice using a disposable pestle in 0.5 mL of RNAiso (Takara; Tokyo, Japan), and samples were added to 0.1 mL of chloroform centrifuged at 12,000× *g* for 15 min at 4 °C. The aqueous layer was collected, mixed with 0.25 mL of isopropanol, and centrifuged at 12,000× *g* for 15 min at 4 °C. The supernatant was discarded, leaving only the pellet that was then washed with 70% ethanol and dissolved in 50 µL of diethyl pyrocarbonate-treated water. The isolated RNA samples were synthesized with cDNA using a Prime Script 1st strand cDNA Synthesis Kit according to the manufacturer’s instructions (Takara).

After synthesis, qRT-PCR was assessed using cDNA samples by the CFX384 TouchTM Real-Time PCR detection system (Bio-Rad Laboratories; Contra Costa, CA, USA). Three hundred nanograms of cDNA samples, 5 μL of SYBR premix (Takara), 0.4 μM forward, and reverse primers (listed in Table S1) were mixed, then threshold cycle numbers were determined using CFX Manager^TM^ software (Version 2.1; Bio-Rad Laboratories).

### 4.4. Enzyme-Linked Immunosorbent Assay (ELISA)

NADP/NADPH^+^ ratio (ab65349; Abcam, Cambridge, UK), SOD activity (706002; Cayman, Ann Arbor, MI, USA), and total glutathione (703002; Cayman Chemical, Ann Arbor, MI, USA) in the gastrocnemius muscle of each group were determined using the appropriate kit, following the manufacturer’s instructions.

### 4.5. Immunohistochemistry (3,3-Diaminobenzidine: DAB)

Tissue blocks of paraffin-embedded gastrocnemius muscle were cut into 7 µm-thick sections, placed on a coated slide, and cooked at 45 °C for 24 h. The Slides with attached gastrocnemius muscle tissue were deparaffinized and incubated in normal animal serum to block antibody nonspecific binding. After blocking, primary antibodies were incubated at 4 °C (listed in Table S2), then treated with biotinylated secondary antibodies using an ABC kit (Vector Laboratories, Burlingame, CA, USA), loaded for 1 h at room temperature. Slides were reacted with DAB substrate for up to 20 min, then mounted with a coverslip and DPX mounting solution (Sigma-Aldrich, St. Louis, MO, USA). Images were assessed using a light microscope (Olympus, Tokyo, Japan), and the intensity of the brown color was quantified using ImageJ software (Version 1.53e, NIH, Bethesda, MD, USA).

### 4.6. Hematoxylin and Eosin (H&E) Staining

H&E staining was used for determining the mean cross-sectional area (CSA) of gastrocnemius and soleus muscle fiber changes. The muscle tissue slides were loaded with hematoxylin (DAKO, Glostrup, Denmark) for 1 min, then rinsed using distilled water for 10 min, and incubated in eosin Y solution (Sigma-Aldrich, St. Lois, MO, USA) for 1 min at room temperature. The completed slides observed under a light microscope (Olympus). The mean CSA of muscle fiber was measured by ImageJ (Version 1.53e, NIH, Bethesda, MD, USA). Histological analyses were conducted in a blinded manner, and three operators conducted at least three replicates of each analysis.

### 4.7. Statistical Analysis

To validate the significance of the differences among the mice, we performed a Kruskal–Wallis test for comparisons of the four groups, followed by a Mann–Whitney U test as a post hoc test. This study was validated using an unpaired *t*-test. All results are presented as mean ± Standard deviation, and statistical significance was set at *p* < 0.05. All statistical analyses were performed using SPSS version 22 (IBM Corporation; Armonk, NY, USA), and the means denoted by a different letter indicate significant intergroup differences.

## Data Availability

Data is contained within the article or supplementary material.

## Supplementary Materials

The following are available online. Figure S1: FO reduced muscle proteolysis through IGF-1 of soleus in a muscle atrophy induced mouse model. Figure S2:FO improved muscle thickness, weight, and fiber size of soleus in a muscle atrophy induced mouse model, Table S1: List of primer for qRT-PCR. Table S2: List of antibodies for immunohistochemistry.

## Sample Availability

The samples of compound (FO, GABA and lactate) used in this study were provided by Marine Bioprocess Co. Ltd.
