# Supplementary material for: Fermented Oyster Extract Attenuated Dexamethasone-Induced Muscle Atrophy by Decreasing Oxidative Stress
Source: Molecules. 2021 Nov 25;26(23):7128. doi: 10.3390/molecules26237128 (PMC8658907; doi:10.3390/molecules26237128)
Supplement: Supplementary file 1 [file molecules-26-07128-s001.zip › molecules-1447513-supplementary.pdf]

Fermented Oyster Extract attenuated dexamethasone-induced muscle atrophy by decreasing oxidative stress

Seyeon Oh [1, #], Kyung-A Byun [1, 2, #], Bae-Jin Lee [3], So Young Lee [4], Kuk-Hui Son [4, *] and Kyunghee Byun [1, 2, *]

[1] Functional Cellular Networks Laboratory, Department of Medicine, Graduate School and Lee Gil Ya Cancer and Diabetes Institute, Gachon University College of Medicine, Incheon 21999, Korea; seyeon8965@gmail.com

[2] Department of Thoracic and Cardiovascular Surgery, Gachon University Gil Medical Center, Gachon University, Incheon 21565, Korea; cch624@gilhospital.com

[3] Marine Bioprocess Co., Ltd., Smart Marine BioCenter, Busan 46048, Korea; hansola82@hanmail.net (B.-J.L.); pdc327@hanmail.net (J.-H.P.)

[4] Department of Anatomy and Cell Biology, Gachon University College of Medicine, Incheon 21936, Korea

* Correspondence: dr632@gilhospital.com (K.-H.S.); khbyun1@gachon.ac.kr (K.B.); Tel.: +82-32-460-3666 (K.-H.S.); +82-32-899-6511 (K.B.)

† These authors contributed equally to this work.

Supplementary Figures

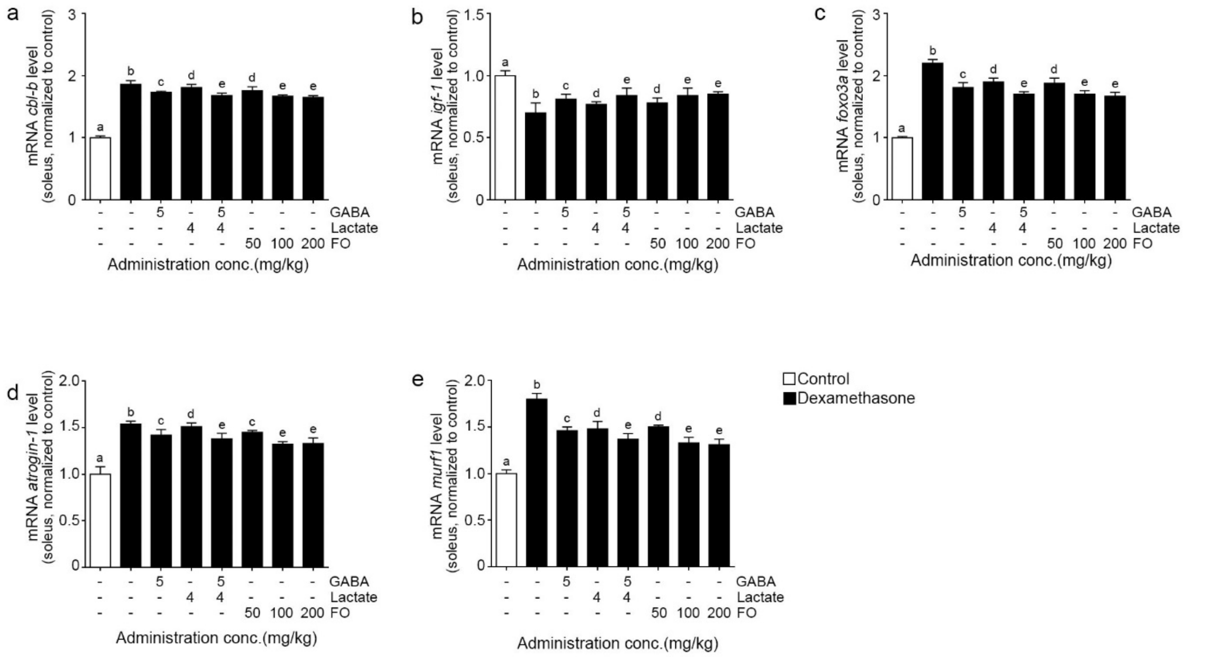

Figure S1. FO reduced muscle proteolysis through IGF-1 of soleus in a muscle atrophy induced mouse model. (**a**) The mRNA levels of *cbl-b* were were increased by Dexa/Saline but decreased by GABA, lactate, GABA+lactate and FO treatement. (**b**) The mRNA levels of *igf-1* were decreased by Dexa/Saline but increased by GABA, lactate, GABA+lactate and FO treatment. (**c**–**e**) The mRNA levels *foxO3a, atrogin-1* and *murf1* were increased by Dexa/Saline but decreased by GABA, lactate, GABA+lactate and FO treatement. a-f; Different letters indicate significant differences among group as determined by multiple comparision (Mann-Whitney U test); $p < 0.05$. cbl-b, Cbl Proto-Oncogene B; FO, fermented oyster extracts; GABA, γ-aminobutyric acid; foxO3a, forkhead box O3; igf-1, insulin like growth factor 1; murf1, Muscle RING-finger protein-1.

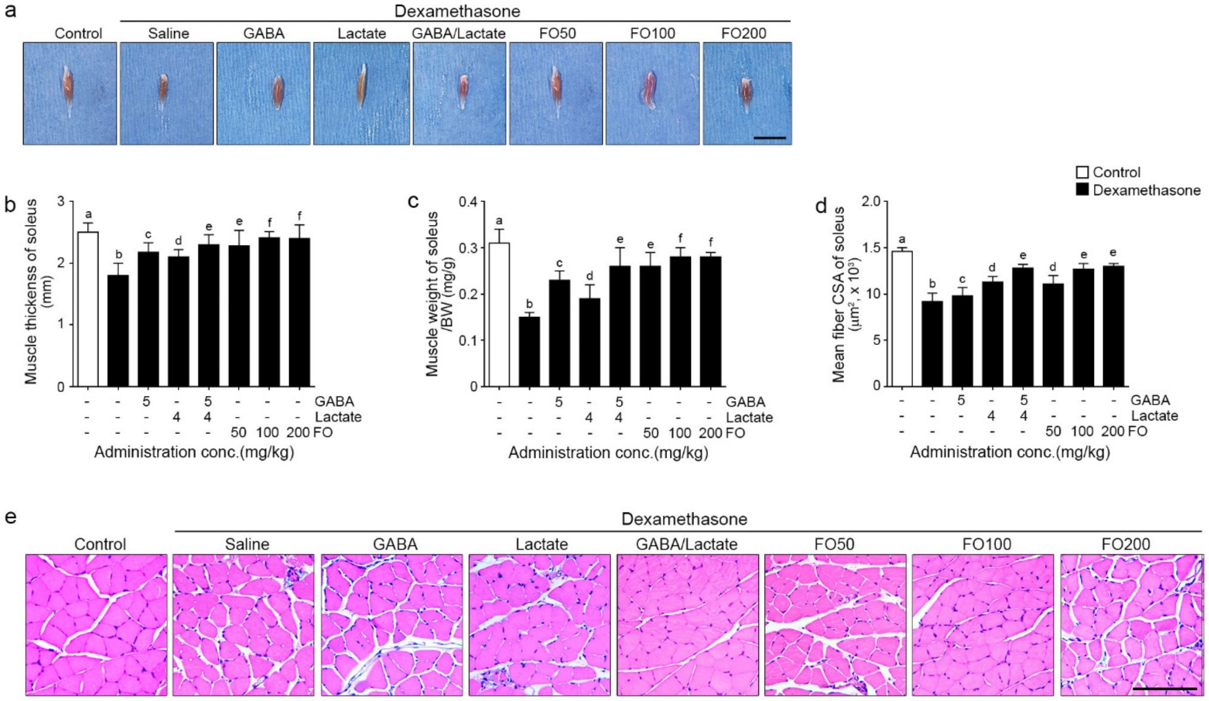

Figure S2. FO improved muscle thickness, weight, and fiber size of soleus in a muscle atrophy induced mouse model. (**a**–**c**) The muscle thckiness of soleus (**a** and **b**) and muscle weigt (**a** and **c**) of soleus were decreased by Dexa/Saline but increased by GABA, lactate, GABA+lactate and FO treatment. (**d** and **e**) The mean CSA of soleus muscle fiber was decreased Dexa/Saline but increased by GABA, lactate, GABA+lactate and FO treatment. a–f; Different letters indicate significant differences among group as determined by multiple comparision (Mann-Whitney U test); $p < 0.05$. BW, body weight; CSA, cross sectional area; FO, fermented oyster extracts; GABA, γ-aminobutyric acid.

Supplementary Tables

Table S1. List of primer for qRT-PCR

Gene		Primers
actb	Forward	5'-ACA AAG CTG TTC AGT GTC TCC A-3′
	Reverse	5'-CTC CGT TTC CAG AAT ACA CAC A-3′
nrf2	Forward	5′-CTC CGT GGA GTC TTC CAT TTA C-3′
	Reverse	5′-ACA CTT CCA GGG GCA CTA TCT A-3′
keap1	Forward	5′-ATG ATC ACA CCG ATG AAT ACC A-3′
	Reverse	5′-CCC CTG CTG CAT AGA TAC AGT T-3′
ho-1	Forward	5′-AGA AGG CTT TAA GCT GGT GAT G-3′
	Reverse	5′-CTT GTT GCG CTC TAT CTC CTC T-3′
cbl-b	Forward	5′-TAT GAC CAG CTC CCT TCA TCT T-3′
	Reverse	5′-ATG AAT TTC TGG TGC AGT CCT T-3′
igf-1	Forward	5′-GAC ATG CCC AAG ACT CAG AAG T-3′
	Reverse	5′-ACT TCC TTT CCT TCT CCT TTG C-3′
foxo3a	Forward	5′-ACC TTC GTC TCT GAA CTC CTT G-3′
	Reverse	5′-AGT GTG ACA CGG AAG AGA AGG T-3′
atrogin-1	Forward	5'-GAC ATT CAG AAC AGC AAA ACC A-3′
	Reverse	5'-GCT CCT TCG TAC TTC CTT TGT G-3′
muRF1	Forward	5'-ATC TAG CCT GAT TCC TGA TGG A-3′
	Reverse	5'-ACC ACA GGC TTG GTA AAC ATC T-3′

Table S2. List of antibodies for immunohistochemistry

Antibody name	Company	Immunohistochemistry
NF-κB	Cell signaling	1 : 400
IL-6	abcam	1 : 50
TNF-α	Novos biological	1 : 200
